# Constructing an Intelligent Model Based on Support Vector Regression to Simulate the Solubility of Drugs in Polymeric Media

**DOI:** 10.3390/ph15111405

**Published:** 2022-11-14

**Authors:** Sait Senceroglu, Mohamed Arselene Ayari, Tahereh Rezaei, Fardad Faress, Amith Khandakar, Muhammad E. H. Chowdhury, Zanko Hassan Jawhar

**Affiliations:** 1 Faculty of Pharmacy, Ege University, Izmir 35040, Turkey; 2 Department of Civil and Architectural Engineering, Qatar University, Doha 2713, Qatar; 3 Technology Innovation and Engineering Education Unit (TIEE), Qatar University, Doha 2713, Qatar; 4 Neuroscience Research Center, Shiraz University of Medical Sciences, Shiraz 71348, Iran; 5 Department of Business, Data Analysis, The University of Texas Rio Grande Valley (UTRGV), Edinburg, TX 78539, USA; 6 Department of Electrical Engineering, Qatar University, Doha 2713, Qatar; 7 Department of Medical Laboratory Science, College of Health Science, Lebanese French University, Erbil 44001, Kurdistan Region, Iraq

**Keywords:** solubility temperature, drug, polymer, support vector regression, kernel type, tuning techniques

## Abstract

This study constructs a machine learning method to simultaneously analyze the thermodynamic behavior of many polymer–drug systems. The solubility temperature of Acetaminophen, Celecoxib, Chloramphenicol, D-Mannitol, Felodipine, Ibuprofen, Ibuprofen Sodium, Indomethacin, Itraconazole, Naproxen, Nifedipine, Paracetamol, Sulfadiazine, Sulfadimidine, Sulfamerazine, and Sulfathiazole in 1,3-bis[2-pyrrolidone-1-yl] butane, Polyvinyl Acetate, Polyvinylpyrrolidone (PVP), PVP K12, PVP K15, PVP K17, PVP K25, PVP/VA, PVP/VA 335, PVP/VA 535, PVP/VA 635, PVP/VA 735, Soluplus analyzes from a modeling perspective. The least-squares support vector regression (LS-SVR) designs to approximate the solubility temperature of drugs in polymers from polymer and drug types and drug loading in polymers. The structure of this machine learning model is well-tuned by conducting trial and error on the kernel type (i.e., Gaussian, polynomial, and linear) and methods used for adjusting the LS-SVR coefficients (i.e., leave-one-out and 10-fold cross-validation scenarios). Results of the sensitivity analysis showed that the Gaussian kernel and 10-fold cross-validation is the best candidate for developing an LS-SVR for the given task. The built model yielded results consistent with 278 experimental samples reported in the literature. Indeed, the mean absolute relative deviation percent of 8.35 and 7.25 is achieved in the training and testing stages, respectively. The performance on the largest available dataset confirms its applicability. Such a reliable tool is essential for monitoring polymer–drug systems’ stability and deliverability, especially for poorly soluble drugs in polymers, which can be further validated by adopting it to an actual implementation in the future.

## 1. Introduction

Enhancing the deliverability of poorly water-soluble solid drugs has always been challenging for researchers engaged in pharmaceutical synthesis [1,2]. Various strategies, including applying nanotechnology [3], nanosuspension [4], nanomedicine [5], solid dispersion [6], surfactants in solid dispersion [7], mesoporous silica material [8], and diverse solution media (such as supercritical [9,10], ionic [11], eutectic [12], the polymer [13]) have already been tried in the field of drug synthesis/formulation. Utilizing the amorphous form of drugs instead of their crystalline structures (i.e., ASD: amorphous solid dispersions) has also gained tremendous attention as a practical path to improve the deliverability and dissolution rate of poorly soluble drugs [14,15]. Dispersing and stabilizing the amorphous form of drugs in polymeric carriers is a well-established class of this ASD scenario [16,17,18,19]. Molecularly dispersing amorphous drugs in polymeric carriers with a higher glass transition temperature (GTT) increases the mixture’s GTT [20], enhances apparent solubility [21], and improves thermodynamic stability [22]. Some researchers also claimed that polymeric carrier selection is the most critical factor in the ASD scenario [23]. Furthermore, drug–polymer miscibility is likely the most important influential factor in achieving amorphous solid dispersion stability [21].

Therefore, reliable insights into drug solubility in polymeric carriers are necessary for successfully implementing the ASD scenario [22,24,25,26,27,28]. The polymer–drug solubility curve is also significant for selecting appropriate polymers for the ASD process and determining the maximum drug loading in the polymeric carrier without the risk of crystallization [28]. Indeed, the drug stability primarily depends on the drug solubility in polymers at the storage temperature [24,29]. To reach a thermodynamically stable formulation, a drug loading in a polymeric carrier should be smaller or at least equal to the drug solubility in the polymer at the storage temperature [24]. In addition, the drug solubility in polymeric media is of interest in the design of pharmaceutical formulation and purification equipment [30]. The drug solubility in polymers is determined by measuring the temperature and the solution dosage at a system equilibrium state [25]. Experimental measurement of the drug solubility is a well-established method in this regard [31,32]. Laboratory measurements of the drug solubility in polymeric carriers is a challenging task due to the unavailability of the standard procedure [25], the high viscosity of polymers [24,33], the difficulty of reaching the equilibrium condition [28], and the consuming time [27].

Therefore, researchers suggested several correlations to estimate the solubility temperature of ibuprofen and naproxen in Soluplus [24] and the miscibility of curcumin in polyvinyl pyrrolidone, hydroxypropyl methylcellulose, and polyethylene glycol [34]. Knopp et al., developed a model utilizing statistical analysis to compare the effect of preparation methods on indomethacin-Polyvinylpyrrolidone solubility curves [35]. The solubility parameter (originally introduced by Hildebrand [36] and then modified by Hansen [37]) is another approach for theoretically determining drug–polymer miscibility. Recently, Mamidi and Rohera constructed the thermodynamic phase diagram of drug–polymer systems using the melting-point depression data, Gordon–Taylor equation, and the Flory–Huggins theory [21]. Such a correlation is only valid for an investigated polymer–drug medium and potentially presents high uncertainty for other polymer–drug systems. To the authors’ best knowledge, the literature has constructed no general empirical, intelligent, or mathematical model for approximating a broad range of polymer–drug systems. Building a universal model helps find the best polymer for drug carriers among different candidates.

Consequently, designing a reliable LS-SVR model to estimate the equilibrium behavior of a wide range of drugs in different polymers is the main objective of the current research. Furthermore, it is necessary to engineer the LS-SVR structure systematically. The suggested LS-SVR can easily produce the solubility curve of a drug in the polymer as a function of drug and polymer type and drug load in the polymer. In addition, this study:

(a) Introduces the most general model to estimate the solubility of drugs in polymers.
(b) Saves time and money by replacing experimental analysis with a straightforward model.
(c) Offers a route for choosing the most suitable polymer for the ASD scenario.
(d) Provides the polymer–drug solubility data for monitoring the solution stability.

## 2. Data Collection

An experimental database is needed to develop an LS-SVR model to approximate the solubility temperature of drugs in polymers. This database should include the dependent variable (T_sol_: solubility temperature) and its related independent variables (i.e., polymer and drug type and drug load in polymers). Table 1 concisely reports polymer–drug systems that will be analyzed in the current study. This table also introduces the names of the drug and polymer based on the original reference, the amount of data, and the range of drug load in polymers and solubility temperature.

Since the collected experimental data covers different polymer–drug systems, molecular weights (Mw) of the drug and polymer are selected to help LS-SVRs discriminate between the behavior of various drugs and polymers during the modeling phase.

In summary, the current study includes 16 drugs, 13 polymers, a drug load of 1 to 100 weight percent (wt%) in polymers, and a solubility temperature of 30 to 252.7 °C. It should be mentioned that the composition of polymer–drug mixtures can vary from low (~0 wt%) to high (~100 wt%) dosages of a drug. Indeed, these points indicate the pure polymer and pure drug, respectively. When the drug loading is 100 wt% (i.e., pure drug), the melting temperature is considered as the solubility temperature [25].

The numerical value of experimental data is available in the Supplementary Materials.

### Relevancy Analysis

**Figure 1:**
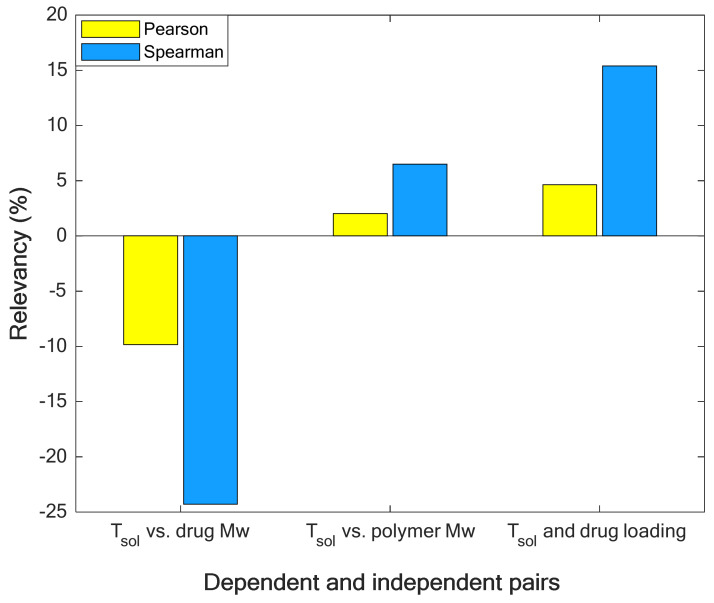
Quantizing the relevancy (strength and direction of relationship) between solubility temperature and its affecting variables.

Two well-known relevancy analysis scenarios, i.e., Spearman [38] and Pearson [38], respectively in Equation (1) are applied to quantize the strength and reveal the direction of relationships between T_sol_ and drug Mw, T_sol_ and polymer Mw, and T_sol_ and drug load in a polymer (Figure 1).

(1) $$\begin{array}{l} \mathrm{Relevancy}=1-\left[\left(6\sum_{n=1}^{N}d_{n}^{2}\right)/\left(N^{3}-N\right)\right]\mathrm{and}\\ \mathrm{Relevancy}={\sum_{n=1}^{N}{\left(IV-IV^{ave}\right)}_{n}\left(T_{sol}^{Exp}-T_{sol}^{ave}\right)}_{n}/\left(\sqrt{\sum_{n=1}^{N}{\left(IV-IV^{ave}\right)}_{n}^{2}}\sqrt{\sum_{n=1}^{N}{\left(T_{sol}^{Exp}-T_{sol}^{ave}\right)}_{n}^{2}}\right) \end{array}$$

where *d* is the difference between the two ranks of observations and *N* is the number of available samples. In addition, *IV* and *IV^ave^* (Equation (2)) show numerical values of the independent variable and their average, and $T_{sol}^{Exp}$ and $T_{sol}^{ave}$ (Equation (3)) are numerical values of the solubility temperature and its average.

(2) $$IV^{ave}=\sum_{n=1}^{N}\left(1/N\right)\times {\left(IV\right)}_{n}$$

(3) $$T_{sol}^{ave}=\sum_{n=1}^{N}\left(1/N\right)\times {\left(T_{sol}^{Exp}\right)}_{n}$$

Figure 1 approves that increasing the molecular weight of drugs decreases the solubility temperature (negative relevancy value shows an indirect relationship [39]). On the other hand, the molecular weight of polymers and drug load in polymers increase the solubility temperature [40] (positive relevancy value shows a direct relationship [39]). In addition, the molecular weight of drugs is the strongest reducing feature (due to its minimum relevancy value [38]), and drug load is the strongest increasing factor (due to its maximum relevancy value [38]) for the solubility temperature.

## 3. LS-SVR Description

Machine learning, deep learning, feature selection, and decision-making techniques have a broad range of applications for implementing either classification or approximation [41] tasks in different fields of daily life, science, and technology [42,43,44]. Support vector machine [45] and its derivation (i.e., least-squares support vector regression [46]) have recently gained great attention. These methods transform the experimental data to a multi-dimensional space utilizing the linear, polynomial, and Gaussian kernels (i.e., *K*) based on Equation (4) [47].

(4) $$K\left(x_{i},x_{j}\right)=\left\{\begin{array}{l} {x_{i}}^{T}x_{j}\;\;\;\;\;\;\;\;\;\;\;\;\;\;\;\;\;\;\;\;\;\;\;\;\;\mathrm{Linear}\;\mathrm{kernel}\\ {\left({x_{i}}^{T}\;x_{j}\;+\;t\right)}^{d}\;\;\;\;\;\;\;\;\;\;\mathrm{Polynomial}\;\mathrm{kernel}\\ e^{-\;{\left\|x_{i}-\;x_{j}\right\|}^{2}/2\sigma^{2}}\;\;\;\;\;\;\;\;\;\;\;\mathrm{Gaussian}\;\mathrm{kernel} \end{array}\right.$$

where *T* is transpose operation, *t*, *d*, and $\sigma^{2}$ are kernel-related parameters.

In the transformed space, it is possible to linearly relate independent and independent variables by Equation (5). *w* (i.e., weights) and *b* (bias) are adjustable parameters of LS-SVR method [48].

(5) $$g\left(x_{i}\right)\;=\;w^{T}\;\varphi \left(x_{i}\right)\;+b\;\;\;\;\;\;\;\;\;\;\;\;\;i=1,\;2,\;\ldots ,N$$

Then, a combination of simulated annealing and simplex methods tries to adjust weights and biases through solving the following optimization algorithm [48].

(6) $$\left\{\begin{array}{l} Minimize\;\;\;\;\;\;\;OF\left(w,e\right)=\frac{1}{2}w^{T}\;w+\frac{\gamma }{2}\sum_{n=1}^{N}e_{n}^{2}\\ e_{n}={\left(T_{sol}^{Exp}\right)}_{n}-w^{T}\;\varphi \left(x_{n}\right)-b\;\;\;\;\;n=1,2,\ldots ,N \end{array}\right.$$

## 4. Results and Discussions

This study aims to determine the best kernel type (Gaussian, polynomial, and linear) and tuning method (leave-one-out and 10-fold cross-validations) of the LS-SVR by trial and error. Hence, it is necessary to adjust the coefficients of the LS-SVR with different kernel types by the leave-one-out and 10-fold cross-validations and compare their accuracy to find the highest accurate topology. *MARDP* (mean absolute relative deviation percent, Equation (7)), *MADP* (mean absolute deviation percent, Equation (8)), *RMSE* (root mean squared error, Equation (9)), and *R*-value (coefficient of determination, Equation (10)) compare the accuracy of different LS-SVR topologies [49] and help find the best one.

(7) $$MARDP={\sum_{n=1}^{N}\left(100/N\right)\times \left(\left|T_{sol}^{Exp}-T_{sol}^{LS-SVR}\right|/T_{sol}^{Exp}\right)}_{n}$$

(8) $$MADP={\sum_{n=1}^{N}\left(100/N\right)\times \left|T_{sol}^{Exp}-T_{sol}^{LS-SVR}\right|}_{n}$$

(9) $$RMSE=\sqrt{{\sum_{n=1}^{N}\left(1/N\right)\times \left(T_{sol}^{Exp}-T_{sol}^{LS-SVR}\right)}_{n}^{2}}$$

(10) $$R=\sqrt{1-\left\{\sum_{n=1}^{N}{\left(T_{sol}^{Exp}-T_{sol}^{LS-SVR}\right)}_{n}^{2}/\sum_{n=1}^{N}{\left(T_{sol}^{Exp}-\;T_{sol}^{ave}\right)}_{n}^{2}\right\}}$$

Here, $T_{sol}^{LS-SVR}$ is the predicted solubility temperature by the LS-SVR.

### 4.1. Constructing the LS-SVR Model

Developing any knowledge-based model needs at least two data collections, i.e., training and testing [50]. Therefore, it is necessary to randomly divide the experimental data into training and testing collections. The former collection is applied to regulate the LS-SVR parameters (known as an LS-SVR training stage). On the other hand, the latter collection is used to appraise the generalization ability of the trained LS-SVR in some unseen situations. This study uses a ratio = 85/15 to allocate the experimental data into the training and testing collections, respectively. Table 2 reports the highest accurate predictions obtained by the LS-SVR models trained by the leave-one-out and 10-fold cross-validation (CV) techniques. The performance of LS-SVRs in the training and testing stages (and the combination of two phases, i.e., overall) has been measured using the aforementioned statistical criteria. The second column of these tables also introduces kernel functions’ adjusted coefficients (t, d, and σ^2^) and regularization parameter (γ).

The reported results in Table 2 approve that LS-SVRs equipped with the Gaussian and linear kernel functions are the highest and lowest accurate models, respectively.

### 4.2. Selecting the Best LS-SVR

Although the previous results showed that the Gaussian function provides the LS-SVR with a higher prediction accuracy than the other kernel types, it is better to demonstrate this finding by helping the ranking analysis. This analysis uses Equation (11) to calculate the average rank of each LS-SVR over the four statistical indexes (MARDP, RMSE, MADP, and *R*-value) [38]. Since ranking analyses have been separately conducted for the training and testing stages (and their combination), it is possible to sort LS-SVRs in different modeling phases.

(11) $$\mathrm{Rank}=\mathrm{round}\left\{{\sum_{i=1}^{4}\mathrm{rank}\left(\mathrm{index}\right)}_{i}/4\right\}$$

#### 4.2.1. Selecting the Best Kernel Function

**Figure 2:**
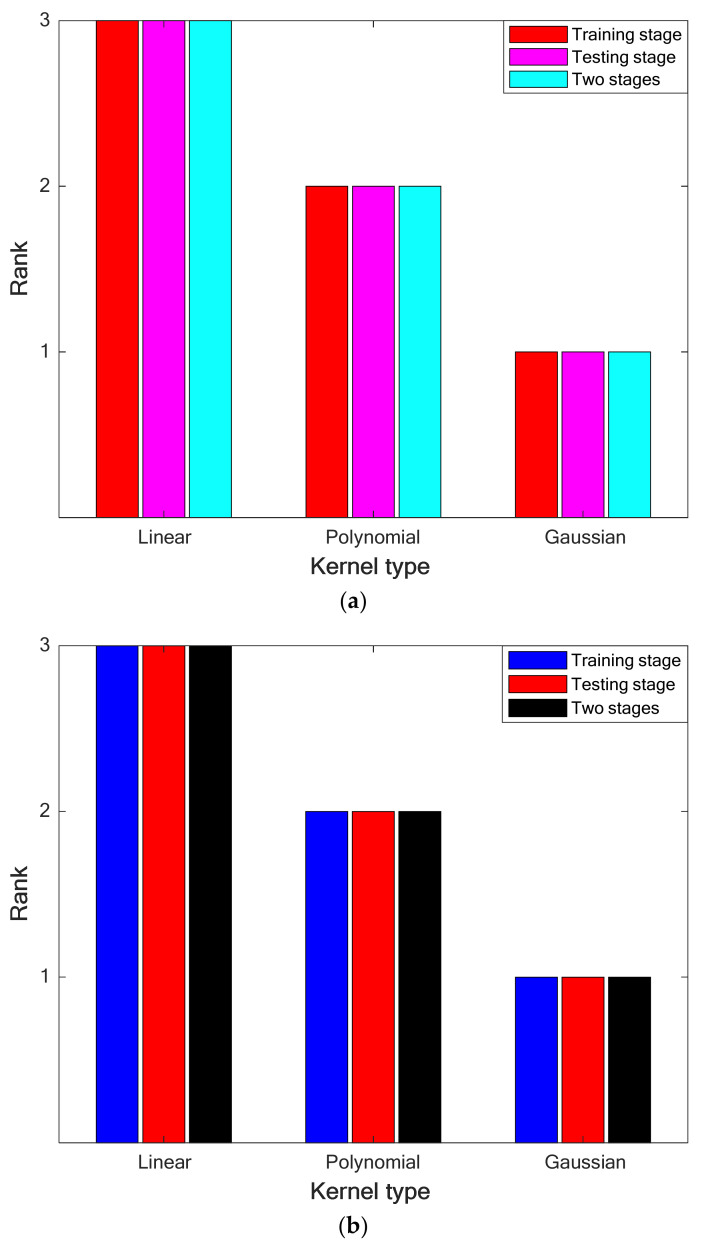
(**a**). Performing the ranking analysis on the performance of LS-SVRs with different kernel types (leave-one-out CV). (**b**). Performing the ranking analysis on the performance of LS-SVRs with different kernel types (10-fold CV).

Results of performing the ranking analysis on the priority of LS-SVRs with different kernel functions have been depicted in Figure 2a,b for the leave-one-out CV and 10-fold cross-CV. Thus, LS-SVRs with the Gaussian kernel function achieve the first ranking in the training/testing stages and their combination.

#### 4.2.2. Selecting the Best Tuning Technique

**Figure 3:**
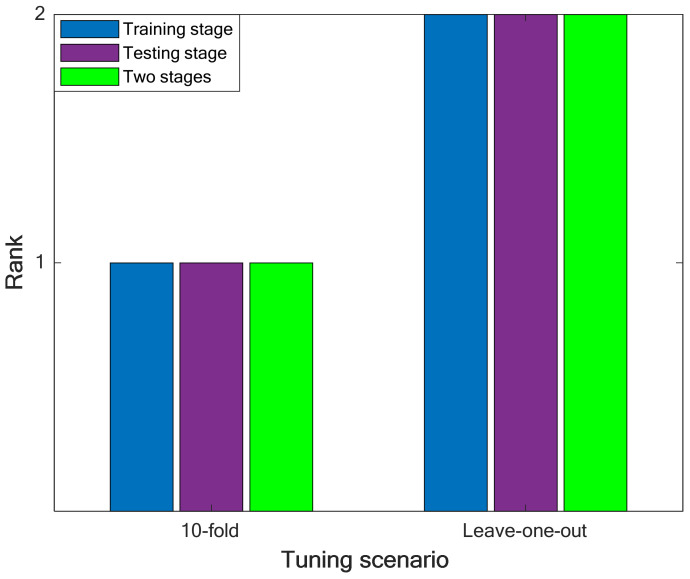
Performing the ranking analysis on the best results of the leave-one-out and 10-fold cross-validation scenarios.

In this stage, the ranking analysis is applied again to determine the best tuning method of the LS-SVR model. The results of performing the ranking analysis on the priority of leave-one-out and 10-fold CV techniques have been illustrated in Figure 3. It can be concluded that the LS-SVR (with the Gaussian kernel function) trained by the 10-fold cross-validation technique presents the highest accuracy in approximating the solubility temperature of drugs in polymers.

Although it is possible to extend the LS-SVR model to cover other drug–polymer binary systems, the current version of the LS-SVR model can only be applied to simulate phase equilibria of the involved drug–polymer systems (see Table 1).

### 4.3. Monitoring the LS-SVR Performance Using Graphical Analyses

#### 4.3.1. Compatibility between Experimental and Calculated T_sol_

Equation (12) measures the actual difference (AD) between experimental and approximated values of the drug solubility temperature in polymers.

(12) $$AD_{n}={\left(T_{sol}^{Exp}-T_{sol}^{LS-SVR}\right)}_{n}\;\;\;\;\;\;\;\;\;\;\;\;\;\;n=1,2,\ldots ,N$$

It is obvious that these observed differences are better to be as small as possible. However, several issues, including the complexity of a considered process/phenomenon, broad ranges of variables, uncertainty in experimental measurements, and modeling error, often result in observing nonzero AD values.

**Figure 4:**
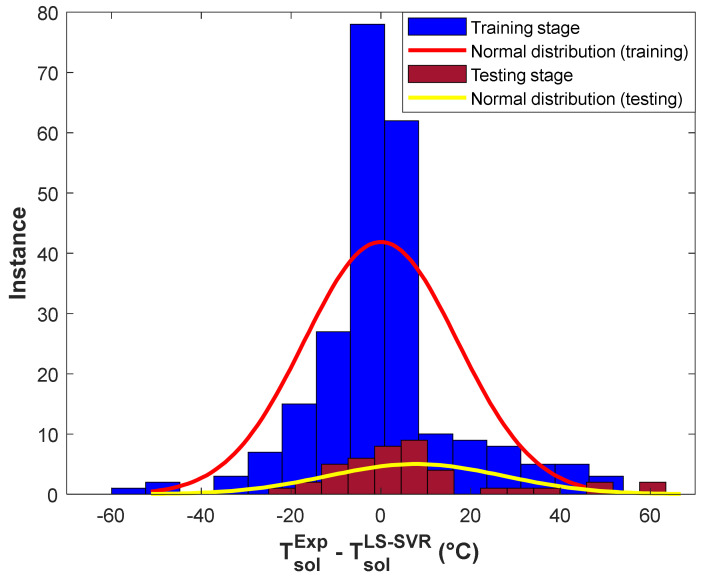
Histogram of residual errors of the structure-tuned LS-SVR in the training and testing stages.

The histogram presentation of the training and testing ADs is shown in Figure 4. This figure indicates that a high percentage of drug solubility temperature in polymers has been estimated with excellent AD values between −20 and 20 °C. In addition, the maximum number of experimental solubility temperatures (~140 training and 30 testing samples) has been computed with the lowest possible AD value (~0 °C). This figure also shows that ~17 experimental solubility samples have been estimated with an AD lower than −40 °C or higher than 40 °C.

This section calculates the average value (AD^ave^) and standard deviation (SD) of presented ADs by the structure-tuned LS-SVR using Equations (13) and (14), respectively [38].

(13) $$AD^{ave}=\sum_{n=1}^{N}\left(1/N\right)\times AD_{n}$$

(14) $$SD=\sqrt{\sum_{n=1}^{N}\left(1/N\right)\times {\left(AD_{n}-AD^{ave}\right)}^{2}}$$

The constructed LS-SVR is able to predict 278 solubility temperatures of 16 drugs in 13 polymers with the promising AD^ave^ = 1.2 °C and SD = 17.7 °C.

#### 4.3.2. Experimental versus Predicted T_sol_ in the Training and Testing Stages

**Figure 5:**
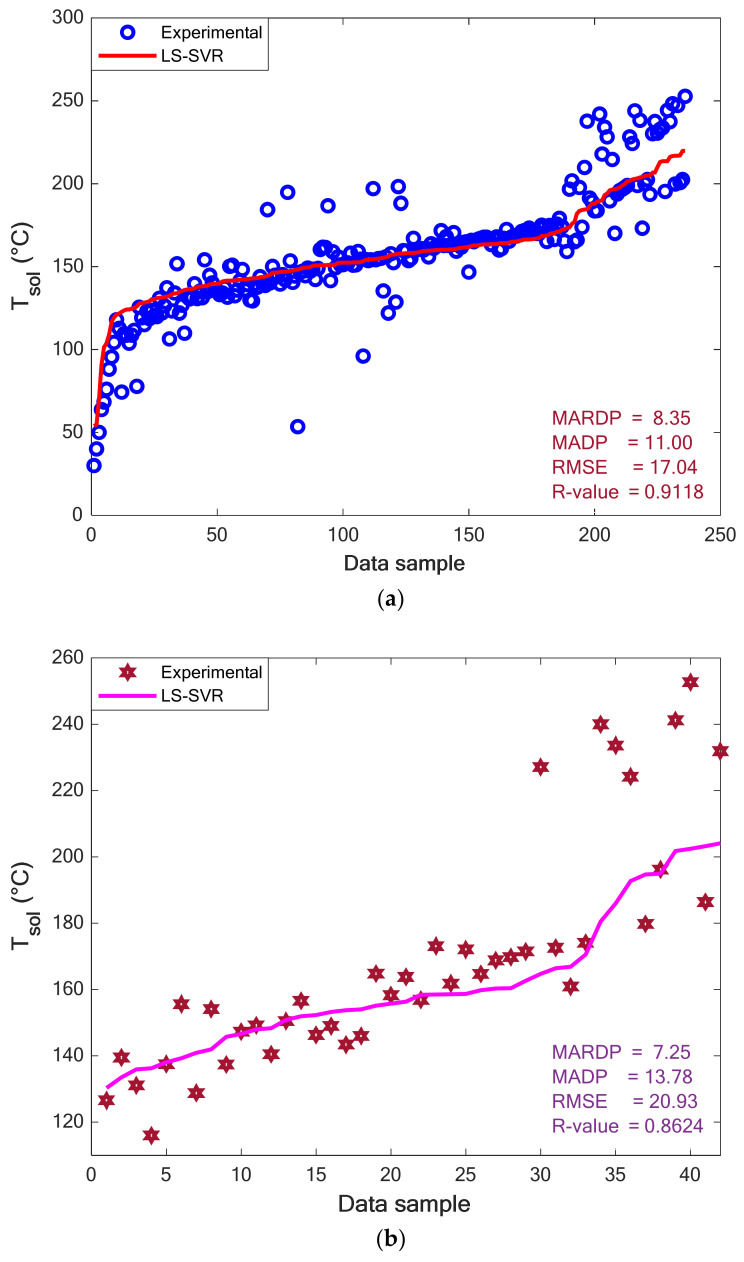
Experimental and modeling values of the solubility temperature in the (**a**) training and (**b**) testing stages.

Figure 5a,b show the value of experimental solubility temperatures in the training and testing stages of the LS-SVR, respectively. Although the testing group possesses a higher scattering level than the training dataset, it can be seen that both groups are scattered enough to make the modeling hard. Despite this relatively high scattering of experimental solubility temperatures, the constructed LS-SVR properly describes the thermodynamic behavior of polymer–drug systems. The excellent performance of the LS-SVR can be more approved by the values of MARDP, MADP, RMSE, and R reported in the figures’ body.

### 4.4. Monitoring the LS-SVR Performance Using Trend Analyses

#### 4.4.1. Evaluating the Impact of Drug Type and Its Load in Polymer on the T_sol_

**Figure 6:**
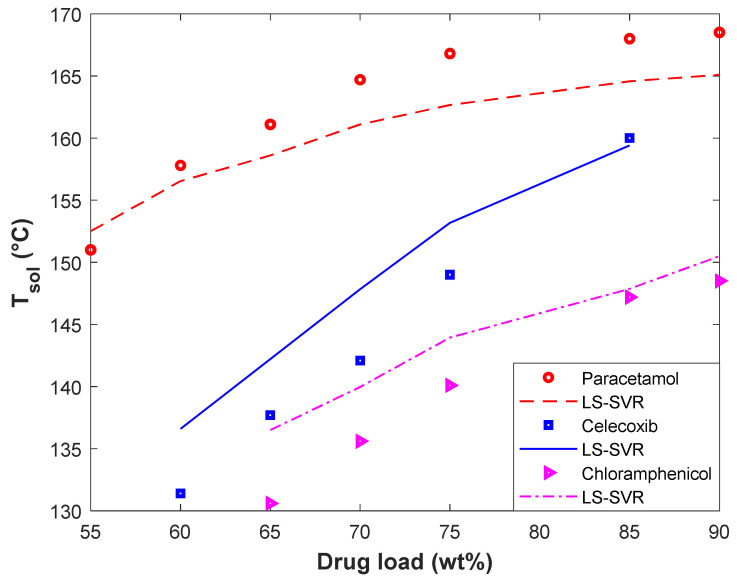
The effect of drug type and drug load in polyvinyl acetate on the solubility temperature.

The effect of the drugs’ type (i.e., Paracetamol, Celecoxib, and Chloramphenicol) and their loading in Polyvinyl Acetate on the solubility temperature has been depicted in Figure 6. This figure presents experimental as well as modeling values of T_sol_ versus drug loading. It can be concluded from these profiles that the drug loading in polymers increases the solubility temperature. Increasing the solubility temperature with increasing the drug loading in the polymer is related to the drug–polymer interactions [26]. It is better to recall that the relevancy analyses also anticipated the increasing impact of drug loading in polymers on the solubility temperature (see Section 2). In addition, the drug type also affects the solubility temperature of drugs in a polymer. It is obvious that changing the solute type changes the interaction between the drug–polymer and the observed solubility temperature.

From the modeling perspective, the LS-SVR successfully describes the thermodynamic behavior of different polymer–drug systems and discriminates among them. Furthermore, the developed model correctly identifies the direct effect of the drug load on the solubility temperature. The excellent agreement between experimental and predicted solubility temperatures can be more justified by the MARDP of 1.55 (Polyvinyl Acetate-Paracetamol), 2.9 (Polyvinyl Acetate-Celecoxib), and 2.59 (Polyvinyl Acetate-Chloramphenicol).

#### 4.4.2. Evaluating the Impact of Polymer Type on the T_sol_

**Figure 7:**
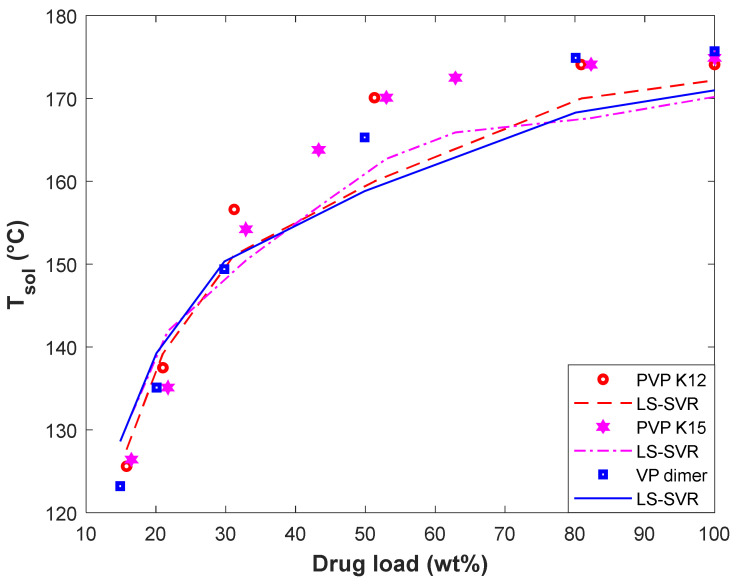
The effect of polymer type and D-Mannitol load on the solubility temperature.

The variation of solubility temperature of D-Mannitol in PVP K12, PVP K15, and VP dimer has been plotted in Figure 7. This figure covers both experimental and modeling T_sol_-drug load profiles. It can be viewed that the PVP K12/D-Mannitol and VP dimer/D-Mannitol systems have the highest and lowest solubility temperature, respectively. The acceptable performance of the LS-SVR for predicting the equilibrium behavior of polymer/D-Mannitol systems can be approved by the observed MARDP value of 1.65 (PVP K12/D-Mannitol), 2.84 (PVP K15/D-Mannitol), and 2.34 (VP dimer/D-Mannitol).

In addition, both the experimental data and LS-SVR curves in Figure 7 show that the effect of the polymer type on solubility temperature is minor. We recall from Figure 1 that the molecular weight of polymers has a minor effect on the drug solubility temperature. On the other hand, PVP K12, PVP K15, and VP dimer are polyvinyl pyrrolidone-based polymers with different molecular weights. Therefore, it is expected that the solubility temperature of D-Mannitol in these polymers is almost equal.

### 4.5. Checking the Data Validity

The experimental data may contain different quantities of noise, outliers, and wrong measurements. Human mistakes, instrument defects, and incorrect calibration are the main cause of this data uncertainty. It seems necessary to check the uncertainty level in the analyzed drug–polymer equilibrium samples. Hence, the last analysis of this study utilizes the leverage method to check the experimental database validity. This method uses the standard residual (SR, Equation (15)) against the Hat index (Equation (16)) graph to find both valid and problematic samples [38].

(15) $$SR_{n}=AD_{n}/SD\;\;\;\;\;\;\;\;\;\;n=1,2,\ldots ,N$$

(16) $$Hatindex=MIV{\left(MIV^{T}\;MIV\right)}^{-1}\;MIV^{T}\;\;\;\left\{\begin{array}{l} MIV:Matrix\;of\;independent\;variables\\ T:\;Transpose\;\;operator \end{array}\right.$$

The samples outside the region confined by critical leverage (CL, Equation (17)) and SR = ±3% are outliers, and all others are valid [51].

(17) $$CL=3\times \left(NIV+1\right)/N\;\;\;\;\;NIV:Number\;of\;independent\;variables$$

**Figure 8:**
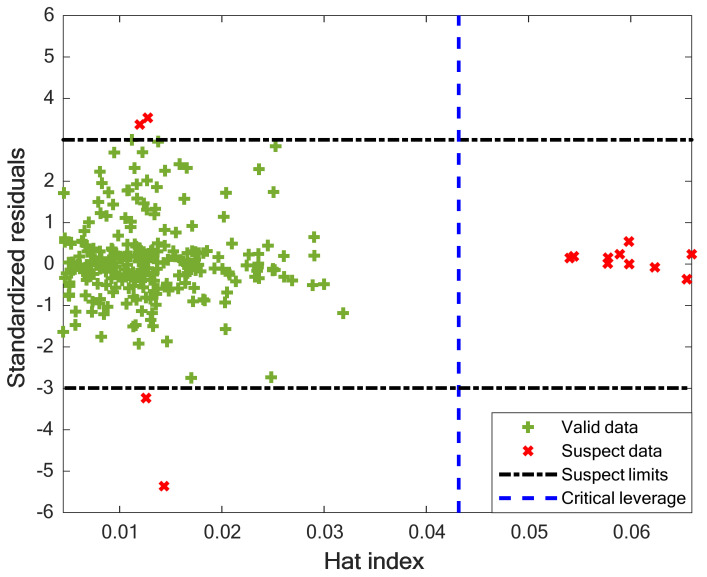
William’s graph to identify outlier as well as valid samples.

Figure 8 reveals that 264 valid and 14 problematic samples exist among the 278 experimental datasets. Indeed, 95% of the experimental databank is valid, and only 5% may be outliers. Since the analyzed experimental samples related to drug–polymer equilibrium behavior are almost valid, the extracted LS-SVR model from this databank is also reliable.

## 5. Conclusions

This study successfully simulated the solubility temperature of 16 drugs in 13 polymeric media by a machine learning technique. The kernel type and tuning method of the LS-SVR have been well-tuned by a systematic combination of statistical and ranking analyses. The results concluded that it is better to equip the LS-SVR with the Gaussian kernel function and adjust its parameters with the 10-fold cross-validation method. The designed LS-SVR predicted the solubility of 278 polymer–drug samples with the MARDP = 8.18, MADP = 11.42, RMSE = 17.69, and R = 0.9037. Since the performance of the LS-SVR model has been validated by the graphical and trend analyses and the utilized experimental database includes 95% valid samples, the suggested model can be readily applied to estimate the solubility temperature of drugs in polymeric media. The relevancy analysis and experimental/modeling profile clarified that the drug solubility temperature in polymers increases by increasing the drug load and the polymer’s molecular weight. On the other hand, the drug’s molecular weight has an inverse relationship with the solubility temperature. This study successfully applied the LS-SVR method to simulate the solubility of different drugs in nonionic polymers. Using machine learning models to estimate the drug solubility in ionic polymers is a good idea to continue this preliminary research study.

## Figures and Tables

**Table 1:** Summary of the literature data for the solubility of drugs in polymers.

Drug Name	Polymer Name	Drug Load (wt%)	T_sol_ (°C)	N	Ref.
Sulfadiazine, Sulfadimidine, Sulfamerazine, Sulfathiazole	PVP, Soluplus	1–100	146.7–252.7	56	[22]
Acetaminophen, Ibuprofen, Ibuprofen Sodium, Itraconazole, Naproxen, Nifedipine	PVP/VA, Soluplus	20–100	30.0–198.3	56	[24]
D-Mannitol, Indomethacin, Nifedipine	PVAc, PVP K12, PVP K15, PVP K25, PVP/VA, VP dimer	5–100	88.2–175.7	59	[25]
Celecoxib, Chloramphenicol, Paracetamol	PVAc, PVP, Soluplus	8–95	95.5–168.5	53	[26]
Celecoxib, Chloramphenicol, Felodipine, Indomethacin, Paracetamol	PVP K17, PVP/VA 335, PVP/VA 535, PVP/VA 635, PVP/VA 735	60–95	132–172	35	[27]
D-Mannitol, Indomethacin, Nifedipine	PVP K15, PVP/VA	22.2–100	108.7–172.1	19	[28]

N: Amount of data. PVAc: Polyvinyl Acetate. PVP: Polyvinylpyrrolidone. PVP/VA: Polyvinylpyrrolidone/vinyl acetate copolymers. Soluplus: Polyvinyl caprolactam-PVAc-polyethylene glycol graft copolymer. T_sol_: Solubility temperature. VP dimer: 1,3-bis[2-pyrrolidone-1-yl] butane.

**Table 2:** The best results obtained by LS-SVRs with different kernel types (leave-one-out CV and 10-fold CV).

The Best Results Obtained by LS-SVRs with Different Kernel Types (Leave-One-Out CV)						
Kernel Type	Tuned Parameters	Group	MARDP	MADP	RMSE	*R*-Value
Linear	γ = 0.07673	Training	19.15	25.15	35.81	0.1726
		Testing	27.73	29.74	43.63	−0.1032
		Overall	20.44	25.84	37.10	0.1161
Polynomial	γ = 1.83 × 10^7^ t = 2.19796 d = 5	Training	10.50	13.73	20.02	0.8538
		Testing	11.20	15.79	22.68	0.7738
		Overall	10.61	14.04	20.44	0.8386
Gaussian	γ = 1.66181 σ^2^ = 0.00765	Training	8.44	11.14	16.99	0.9234
		Testing	9.70	15.35	18.92	0.8455
		Overall	8.63	11.78	17.30	0.9148
**The Best Results Obtained by LS-SVRs with Different Kernel Types (10-Fold CV)**						
**Kernel Type**	**Tuned Parameters**	**Group**	**MARDP**	**MADP**	**RMSE**	***R*-Value**
Linear	γ = 0.06677	Training	20.52	26.82	37.62	0.1506
		Testing	20.68	20.85	34.09	−0.0893
		Overall	20.54	25.92	37.10	0.1133
Polynomial	γ = 0.01154 t = 7.10159 d = 5	Training	15.68	18.34	28.11	0.6236
		Testing	14.37	19.93	28.19	0.8333
		Overall	15.48	18.58	28.13	0.6661
Gaussian	γ = 1.98636 σ^2^ = 0.01335	Training	8.35	11.00	17.04	0.9118
		Testing	7.25	13.78	20.93	0.8624
		Overall	8.18	11.42	17.69	0.9037

## Data Availability

Data is contained within the article and Supplementary Materials.

## Supplementary Materials

The numerical value of independent and dependent variables of the analyzed experimental data can be downloaded at: https://www.mdpi.com/article/10.3390/ph15111405/s1.
