# Correction: Senceroglu et al. Constructing an Intelligent Model Based on Support Vector Regression to Simulate the Solubility of Drugs in Polymeric Media. *Pharmaceuticals* 2022, *15*, 1405

**DOI:** 10.3390/ph19010152

**Published:** 2026-01-15

**Authors:** Sait Senceroglu, Mohamed Arselene Ayari, Tahereh Rezaei, Fardad Faress, Amith Khandakar, Muhammad E. H. Chowdhury, Zanko Hassan Jawhar

**Affiliations:** 1 Faculty of Pharmacy, Ege University, Izmir 35040, Turkey; 2 Department of Civil and Architectural Engineering, Qatar University, Doha 2713, Qatar; 3 Technology Innovation and Engineering Education Unit (TIEE), Qatar University, Doha 2713, Qatar; 4 Neuroscience Research Center, Shiraz University of Medical Sciences, Shiraz 71348, Iran; 5 Department of Business, Data Analysis, The University of Texas Rio Grande Valley (UTRGV), Edinburg, TX 78539, USA; 6 Department of Electrical Engineering, Qatar University, Doha 2713, Qatar; 7 Department of Medical Laboratory Science, College of Health Science, Lebanese French University, Erbil 44001, Kurdistan Region, Iraq

**References Correction**

In the published manuscript [1], Reference 3 was incorrectly listed as ‘Zheng, J.; Long, X.; Chen, H.; Ji, Z.; Shu, B.; Yue, R.; Liao, Y.; Ma, S.; Qiao, K.; Liu, Y.; et al. Photoclick Reaction Constructs Glutathione-Responsive Theranostic System for Anti-Tuberculosis. *Front. Mol. Biosci.* **2022**, *9*, 845179.’. It has been correctly replaced with [2].

Reference 5 was incorrectly listed as ‘Yang, W.; Liu, W.; Li, X.; Yan, J.; He, W. Turning chiral peptides into a racemic supraparticle to induce the self-degradation of MDM2. *J. Adv. Res.* 2022; *in press*.’. It has been correctly replaced with [3].

Reference 13 was incorrectly listed as ‘Kazemi, M.; Emami, J.; Hasanzadeh, F.; Minaiyan, M.; Mirian, M.; Lavasanifar, A. Pegylated multifunctional pH-responsive targeted polymeric micelles for ovarian cancer therapy: Synthesis, characterization and pharmacokinetic study. *Int. J. Polym. Mater. Polym. Biomater.* **2021**, *70*, 1012–1026.’. It has been correctly replaced with [4].

Reference 19 was incorrectly listed as ‘Zhang, X.; Qu, Y.Y.; Liu, L.; Qiao, Y.N.; Geng, H.R.; Lin, Y.; Xu, W.; Cao, J.; Zhao, J.Y. Homocysteine inhibits pro-insulin receptor cleavage and causes insulin resistance via protein cysteine-homocysteinylation. *Cell Rep.* **2021**, *37*, 109821.’. It has been correctly replaced with [5].

Reference 22 was incorrectly listed as ‘Caron, V.; Hu, Y.; Tajber, L.; Erxleben, A.; Corrigan, O.I.; McArdle, P.; Healy, A.M. Amorphous solid dispersions of sulfonamide/soluplus® and sulfonamide/PVP prepared by ball milling. *AAPS PharmSciTech* **2013**, *14*, 464–474.’. It has been correctly replaced with [6].

Reference 29 was incorrectly listed as ‘Wang, X.H.; Xu, S.; Zhou, X.Y.; Zhao, R.; Lin, Y.; Cao, J.; Zang, W.D.; Tao, H.; Xu, W.; Li, M.Q.; et al. Low chorionic villous succinate accumulation associates with recurrent spontaneous abortion risk. *Nat. Commun.* **2021**, *12*, 3428.’. It has been correctly replaced with [7].

Reference 31 was incorrectly listed as ‘Qu, Y.; Zhao, R.; Zhang, H.; Zhou, Q.; Xu, F.; Zhang, X.; Xu, W.H.; Shao, N.; Zhou, S.X.; Dai, B.; et al. Inactivation of the AMPK–GATA3–ECHS1 Pathway Induces Fatty Acid Synthesis That Promotes Clear Cell Renal Cell Carcinoma Growth. *Cancer Res.* **2020**, *80*, 319–333.’. It has been correctly replaced with [8].

Reference 32 was incorrectly listed as ‘Wang, D.; Wang, F.; Shi, K.H.; Tao, H.; Li, Y.; Zhao, R.; Lu, H.; Duan, W.; Qiao, B.; Zhao, S.M.; et al. Lower Circulating Folate Induced by a Fidgetin Intronic Variant Is Associated with Reduced Congenital Heart Disease Susceptibility. *Circulation* **2017**, *135*, 1733–1748.’. It has been correctly replaced with [9].

Reference 40 was incorrectly listed as ‘Lai, W.F.; Gui, D.; Wong, M.; Döring, A.; Rogach, A.L.; He, T.; Wong, W.T. A self-indicating cellulose-based gel with tunable performance for bioactive agent delivery. *J. Drug Deliv. Sci. Technol*. **2021**, *63*, 102428.’. It has been correctly replaced with [10].

Reference 41 was incorrectly listed as ‘Alibak, A.H.; Khodarahmi, M.; Fayyazsanavi, P.; Alizadeh, S.M.; Hadi, A.J.; Aminzadehsarikhanbeglou, E. Simulation the adsorption capacity of polyvinyl alcohol/carboxymethyl cellulose based hydrogels towards methylene blue in aqueous solutions using cascade correlation neural network (CCNN) technique. *J. Clean. Prod.* **2022**, *337*, 130509.’. It has been correctly replaced with [11].

Reference 42 was incorrectly listed as ‘Song, K.; Wu, D. Shared decision-making in the management of patients with inflammatory bowel disease. *World J. Gastroenterol.* **2022**, *28*, 3092–3100.’. It has been correctly replaced with [12].

Reference 43 was incorrectly listed as ‘Duan, C.; Deng, H.; Xiao, S.; Xie, J.; Li, H.; Zhao, X.; Han, D.; Sun, X.; Lou, X.; Ye, C.; et al. Accelerate gas diffusion-weighted MRI for lung morphometry with deep learning. *Eur. Radiol.* **2022**, *32*, 702–713.’. It has been correctly replaced with [13].

Reference 44 was incorrectly listed as ‘Zou, M.; Yang, Z.; Fan, Y.; Gong, L.; Han, Z.; Ji, L.; Hu, X.; Wu, D. Gut microbiota on admission as predictive biomarker for acute necrotizing pancreatitis. *Front. Immunol.* **2022**, *13*, 988326.’. It has been correctly replaced with [14].

The authors state that the scientific conclusions are unaffected. This correction was approved by the Academic Editor. The original publication has also been updated.
